# Prospecting for Breast Cancer Blood Biomarkers: Death-Associated Protein Kinase 1 (DAPK1) as a Potential Candidate

**DOI:** 10.1155/2020/6848703

**Published:** 2020-05-24

**Authors:** Benjamin Arko-Boham, Bright Afriyie Owusu, Nii Ayite Aryee, Richard Michael Blay, Ewurama Dedea Ampadu Owusu, Emmanuel Ayitey Tagoe, Abdul Rashid Adams, Richard Kwasi Gyasi, Nii Armah Adu-Aryee, Seidu Mahmood

**Affiliations:** ^1^Department of Medical Laboratory Sciences, School of Biomedical and Allied Health Sciences, College of Health Sciences, University of Ghana, Ghana; ^2^Department of Anatomy, School of Biomedical and Allied Health Sciences, University of Ghana, Ghana; ^3^Department of Medical Biochemistry, School of Biomedical and Allied Health Sciences, College of Health Sciences, University of Ghana, Ghana; ^4^Centre of Tropical Medicine and Travel Medicine, Department of Infectious Diseases, Division of Internal Medicine, Academic Medical Centre, University of Amsterdam, Netherlands; ^5^Foundation for Innovative and New Diagnostics (FIND), Geneva, Switzerland; ^6^West African Centre for Cell Biology of Infectious Pathogens, University of Ghana, Legon, Ghana; ^7^Department of Pathology, School of Biomedical and Allied Health Sciences, University of Ghana, Ghana; ^8^Department of Surgery, School of Medicine and Dentistry, College of Health Sciences, University of Ghana, Ghana; ^9^Department of Surgery, Korle-Bu Teaching Hospital, Accra-, Ghana

## Abstract

**Background:**

Breast cancer is the commonest malignancy in women worldwide. It is estimated to affect approximately 1.5 million women annually and responsible for the greatest number of cancer-related mortalities among women. In 2018, breast cancer mortalities stood at 627,000 women representing approximately 15% of all cancer deaths among women. In Ghana, breast cancer is the second leading cause of cancer deaths, with an incidence of 2,900 cases annually; one of eight women with the disease die. This gives impetus to the fight for improved early detection, treatment, and/management. In this light, we investigated the potential of death-associated protein kinase 1 (DAPK1) as a biomarker for breast cancer. As a tumour suppressor, its expression is activated by several carcinogens to influence cellular pathways that result in apoptosis, autophagy, immune response, and proliferation.

**Aim:**

To investigate DAPK1 as a blood biomarker for breast cancer.

**Methods:**

Blood samples of participants diagnosed with breast cancer and healthy controls were collected and processed to obtain serum. Information on age, treatment, diagnosis, and pathology numbers was retrieved from folders. Pathology numbers were used to retrieve breast tissue blocks of patients at the Department of Pathology of the KBTH. Tissue blocks were sectioned and immunohistochemically stained with anti-DAPK1 and counterstained with hematoxylin to determine the DAPK1 expression levels. DAKP1 levels in blood sera were quantified using a commercial anti-DAPK1 ELISA kit. Case and control group means were compared using one-way ANOVA and Chi-square test. Statistical significance was set at *p* ≤ 0.05. *Results and Discussion*. DAPK1 levels were higher in sera and breast tissues of breast cancer patients than controls. The augmented DAPK1 expression can be interpreted as a stress response survival mechanism to remediate ongoing deleterious events in the cells orchestrated by carcinogenesis. In the presence of abundant DAPK1, the proliferative power of cells (both cancerous and noncancerous) is increased. This may explain why high DAPK1 expression strongly associates with aggressive breast cancer phenotypes like the ER-negative breast cancers, especially the triple-negative breast cancers (TNBC) which are the most aggressive, fast-growing, and highly metastatic.

**Conclusion:**

DAPK1 is highly expressed in sera and breast tissues of breast cancer patients than nonbreast cancer participants. The elevated expression of DAKP1 in circulation rather than in breast tissues makes it a candidate for use as a blood biomarker and potential use as therapeutic target in drug development.

## 1. Introduction

The World Health Organisation (WHO) report for 2018 indicates that cancer is the second leading cause of death globally accounting for 9.6 million deaths and further state that nearly 1 in 6 deaths globally is due to cancer with about 70% of these deaths occurring in low- and middle-income countries [1]. Breast cancer is the world's commonest cancer among women. It has been estimated to affect approximately 2.1 million women annually and is responsible for the greatest number of cancer-related mortalities among women. Breast cancer mortalities in the year 2018 alone stood at 627,000 women representing approximately 15% of all cancer deaths among women [2, 3]. Currently, breast cancer prevalence and mortality rates are seeing worryingly increasing trends in women in nearly all regions of the globe. This is a departure from what historically used to be restricted to women in the more developed regions of the world [4]. However, though breast cancer incidence (number of cases per 100,000 women) still remains lower in developing countries overall than in the developed countries, death rates from the disease are higher in developing countries compared to their developed counterparts with [5–7].

The incidence of breast cancer in developing countries in Africa particularly has increased, deviating from earlier reports [8]. The disease in a number of countries has surpassed cervical cancer as the most frequently diagnosed cancer in women in Africa [9, 10] as exampled by reports from Ghana [11, 12], Nigeria [13], and Uganda [14]. From Ghana alone, WHO reported in year 2012 that about 2,000 Ghanaian women were diagnosed with breast cancer, out of which 1,000 (50%) died. Currently, breast cancer has been identified as the second leading cause of cancer deaths in Ghana, with an incidence of 2,900 cases annually and at least one of eight women with the disease dying [2, 15].

The high mortality rates of the disease in developing countries are mostly attributed to late diagnosis and poor access to treatment. To improve breast cancer outcomes and survival, early detection is critical. Biomarkers have proven their usefulness in predicting disease incidence, disease progression, and disease outcomes. Death-associated protein kinase 1 (DAPK1) is part of a group of kinases like DAPK2, DAPK3, DAP kinase-related apoptosis-inducing protein kinase 1 (DRAK1), and DRAK2 [16]. Located on the long arm of chromosome 9, region 3, subregion 4.1, it is a calcium/calmodulin-dependent serine/threonine kinase involved in multiple cellular signaling pathways that trigger death, autophagy, and cell survival [16]. DAPK1 is a protein modulated by calcium through the p53 dependent pathways for the instigation of death signals [17–19]. It encodes structurally unique 160kD protein and is made up of 8 ankyrin repeats and 2 putative P-loop consensus positions. It is a tumour suppressor or candidate tumour suppressor protein [20].

Physical, chemical, and biological carcinogens which stimulate DAPK1 increase the expression of the protein via the activation of the dephosphorylation of ser 308 and p53 through the p14/p19ARF pathway, which results in programmed cell death (apoptosis), autophagy, immune response, and proliferation [21, 22]. The role of DAPK1 may change depending on the cell setting. Cell growth is regulated by DAPK1 through the modulation of TSC1/TSC2 complex formation in the mTOR pathway. Phosphorylation of TSC1 and TSC2 leads to upregulation in cell growth and protein synthesis and thereby ensuring a homeostatic equilibrium between survival and death signaling. However, in p53 mutant cells, the dysfunction of p53 prevents DAPK1 from inducing apoptosis, but rather shifts its function from apoptosis towards activation of growth pathways. Zhao and colleagues [16] have reported that in p53 wild type, DAPK1 regulates apoptosis but changes role in p53 mutant setting and becomes proproliferative, hence its varying expression patterns in different cancers. This report suggests the sensitivity of certain cancers like ovarian and pancreatic cancers to DAPK1 inhibitors. It is therefore worth investigating DAPK1 and its functional relevance in breast cancers. This study focused on the usefulness of DAPK1 as a prognostic blood biomarker for breast cancer and its reliability in the light of chemotherapy.

## 2. Material and Methods

### 2.1. Study Participants

Participants were women clinically diagnosed with breast cancer at Korle-Bu Teaching Hospital (KBTH), Accra, Ghana. Controls were apparently healthy individuals in the same facility and its immediate environs.

### 2.2. Sample Collection Procedure

#### 2.2.1. Blood Samples

Blood (5 ml) was drawn from consented participants at the chemotherapy suite of the Department of Surgery, Korle-Bu Teaching Hospital during regular treatment sessions of patients when patients' veins were punctured using a cannula for drug administration. This was done prior to drug administration to prevent double puncture of the veins of the participants who were prepared by assigned nursing officers. Blood samples were collected into serum separator tubes and allowed to clot at room temperature and centrifuged at 2500 rpm for 20 minutes. The sera were collected into fresh tubes and stored at -20°C until use. In total, 32 breast cancer patients and 32 apparently health women participated in the study as cases and controls, respectively.

#### 2.2.2. Breast Tissue Biopsies

Pathology identification numbers were noted from pathology reports of patients in their hospital folders (patient's clinical records), and their archived breast biopsy tissue blocks and haematoxylin and eosine (H&E) stained tissue sections were tracked and retrieved from the Department of Pathology of the Hospital. Retreived H&E stained tissue slides were screened, and the tumour grades confirmed by a pathologist. The corresponding paraffin-embedded tissue blocks of the H&E stained tissue slides were prepared for sectioning to obtain 4 *μ*m thick tissue sections, which were placed on poly-lysine-coated glass slides.

### 2.3. Enzyme-Linked Immunosorbent (ELISA) Assay

Stored sera were retrieved and allowed to thaw at room temperature. Following manufacturer's instructions, the wells of the ELISA plate precoated with anti-DAPK1 (GenWay ELISA kit, Republic of China) were washed to condition them for the antigen-antibody interactions. 100 *μ*l of 1XPBS was gently dispersed in the wells and shaken gently for 30 seconds, then decanted. Further drying was done using filter paper or pad. Following this, 50 *μ*l of each sample and standards was pipetted into designated ELISA plate wells precoated with anti-DAPK1. The samples were added to horseradish peroxidase-labeled recognition antigens specific to DAPK1 in the wells and incubated at 37°C for 1 hour. TMB substrate solution was added to each well and incubated for 10 minutes; after which, a stop solution was added to each well. The optical density (OD) was measured using a spectrophotometer at 450 nm. DAPK1 concentrations were determined from standard curves.

### 2.4. Tissue Section Treatment and Immunohistochemistry

Tissue sections were dewaxed at 50°C (5 min) and in xylene (5 min). They were subsequently put in decreasing grades of ethanol; absolute, 90%, 70%, 50%, 40%, 25%, and finally into water. Citrate buffer (pH = 6.0) was subsequently used for antigen retrieval. Coplin jars containing 1x citrate buffer were heated to boiling point in a pressure cooker on a hot plate. At boiling point, the pressure cooker was opened, and the hydrated tissue sections were transferred into the coplin jars in the pressure cooker and the lid secured. As soon as the cooker had reached full pressure, the coplin jars and their contents were allowed to boil for 5 minutes after which the heat was turned off and placed in an empty sink. The pressure valve was subsequently released, and cold water was run over the cooker. Once depressurized, the lid was opened and allowed to sit and cool before slides were removed and transferred into distilled water.

To quench endogenous peroxidase activities, the cooled tissue sections were flooded with 3% hydrogen peroxidase solution (H_2_O_2_) for 10 minutes at room temperature. Sections were thoroughly washed thereafter with 1XPBS. Subsequently, bovine serum albumin (BSA) was used for blocking. The sections were covered with BSA for 10 minutes at room temperature and then washed with 1XPBS. Tissue sections were then incubated with the primary anti-DAPK1 solution (Boster Biological Technology Company Limited, Republic of China) for 5 hours at room temperature. The dilution factor for the anti-DAPK1 solution was 1 : 50 with 1XPBS as diluent. Sections were washed with 1XPBS after the incubation period after which they were incubated with a biotinylated secondary antibody for 20 minutes at room temperature. Sections were rinsed again in 1XPBS and treated with horseradish peroxidase-conjugated streptavidin (HRP) (Boster Biological Technology Company limited, Republic of China) solution for 10 minutes at room temperature. DAB substrate solution was then dispensed onto the sections for colour development. Tissue sections were rinsed thoroughly with tap water and counterstained with Mayer's hematoxylin (Sigma-Aldrich, Germany) and mounted with DPX (Gain Chemical company).

### 2.5. Immunohistochemistry Scoring

Scoring was done by two independent experts using an Olympus light microscope (Olympus CX31, model CX31RBSF). The scoring was based on the chromogenic intensity of the DAB reaction on the tissue sections under the microscope. Sections that showed no DAB reaction were scored zero (0). Sections with mild reaction were scored +1, those with moderate staining reaction were scored +2, and those that showed strong reaction scored +3. Thirty-two tissue biopsies corresponding to the 32 recruited breast cancer patients were successfully retrieved, sectioned, and stained. Also, thirty-two (32) breast biopsies that had been confirmed as noncancerous were randomly selected to serve as control (nonbreast cancer) and for comparison. Pictures of the sections were captured using an Olympus digital camera (model DP20) mounted on an Olympus microscope (model BX51 TF).

### 2.6. Statistical Analysis

Statistical analysis was performed using SPSS 23 software. Descriptive statistics were used to summarize the data. Student's *t*-test and one-way ANOVA were used to compare means of grouped quantitative data. Comparison between categorical data was done using Chi-square test. In all analyses, *p* value ≤ 0.05 was considered statistically significant. Values are presented as mean ± SD to two decimal places.

### 2.7. Ethical Approval

The Institutional Review Board (IRB) of the Korle-Bu Teaching Hospital and the Ethical and Protocol Review Committee of the School of Biomedical and Allied Health Sciences, College of Health Sciences, University of Ghana both gave approval for the conduct of the study with clearance numbers STC/IRB/000100/2016 and MD/10550649/AA/5A/2016-2017, respectively.

## 3. Results

### 3.1. Demographics and Clinical Information of Participants

Sixty-four (64) participants were recruited; 32 breast cancer patients (cases) and 32 healthy individuals (controls). All participants were females living within the Greater Accra region of Ghana and its environs. Of the breast cancer cases, 10 were petty traders, 15 were unemployed and 7 were government workers (Table 1). Their mean age was 45 years, and that of the controls was 40 years. Invasive ductal carcinoma was the commonly diagnosed (94%) breast cancer type, and preponderant tumour grade was grade II. Of the 30 diagnosed invasive ductal carcinomas, 8 (25%) had already undergone mastectomy while the remaining 24 (75%) had not.

### 3.2. DAPK1 Expression in Sera

DAPK1 expression in sera of breast cancer patients was significantly higher than in the controls with the values 4.95 (SD = 1.53) ng/ml and 3.49 (SD = 1.72) ng/ml, respectively, and *p* value of 0.039 (Table 2). A further analysis was made by comparing the DAPK1 expression pattern between breast cancer patients already on treatment (*treatment*) and those yet to be placed on treatment (*pretreatment*). Of the 32 cases, 17 were classified “*treatment*” and 15 “*pretreatment*”. From Table 2, although the *treatment* group had slightly elevated serum DAPK1 expression than the *pretreatment* group, the difference was not statistically significant.

### 3.3. DAPK1 Expression in Breast Tissue Biopsies

**Figure 1:**
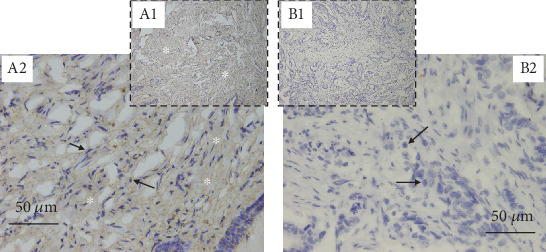
Representative micrographs of immunohistochemically stained breast tissue sections. A1 and B1 are images of breast cancer and nonbreast cancer tissue sections, respectively, captured at ×10 magnification. A2 and B2, respectively, are A1 and B1 captured at higher magnification of ×40. The asterisks (^∗^) represent areas of “*browning*” indicating positive DAB reaction. The black arrows are pointing to the nuclei of cells.

Immunohistochemically stained breast tissue sections were scored by two qualified experts. Regarding the staining intensities, there were no inter-observer differences.  Table 3 and Figure 1 show the DAPK1 expression levels among breast cancer (cases) and nonbreast cancer (controls) biopsies. DAPK1 levels were significantly elevated in breast cancer biopsies compared to nonbreast cancer biopsies (*p* < 0.001).

## 4. Discussion

We have previously reported that DAPK1 was elevated in archived serum samples of breast cancer patients compared to nonbreast cancer individuals and thus the protein was associated with aggressive breast tumour phenotypes in Ghanaians [23]. In furtherance to the earlier report, we present findings from a prospective study, which are confirmatory of our earlier report and strengthen our views on DAPK1 and its dependability as a potential biomarker. We report that DAPK1 expression levels in both sera and breast tissue samples are higher in breast cancer patients than in their nonbreast cancer counterparts.

Physiologically, the expression of DAPK1 is activated by several carcinogens to influence cellular pathways that result in apoptosis, autophagy, immune response, and proliferation [21, 22]. DAPK1 as a tumour suppressor regulates autophagy and apoptosis (mediator of gamma-interferon induced programmed cell death) thereby acting as an important player in the pathway involved in the ER stress-induced apoptosis [24, 25]. Against this backdrop, increased DAPK1 expression is suggestive of possible carcinogenic insults to the cells of the body. Accordingly, breast cancer patients in this present study recorded elevated DAPK1 expression compared to controls. The augmented serum levels of DAPK1 may be an indication of the perturbations within cells of breast cancer patients at the molecular level [23]. This is confirmed by the high levels of the protein in breast tissues of the patients (Table 3 and Figure 1). The augmented DAPK1 expression can be interpreted as a stress response survival mechanism to remediate ongoing deleterious events in the cells orchestrated by carcinogenesis. The body, in its attempts to promote apoptosis and autophagy in the molecularly deranged breast cells, elevates DAPK1 expression. This is a positive action to rid the tissue of the deranged cells. However, the abundance of the protein in the system, though desired, comes with undesired concomitant outcomes which include proproliferative cellular activities. Cancer cells have defective cellular control mechanisms and therefore are characterized by high proliferative and growth rates. In the presence of abundant pro-proliferation DAPK1, the proliferative power of cells (both cancerous and noncancerous) is increased [24]. This may explain why high DAPK1 expression strongly associates with the aggressive breast cancer phenotypes like the ER-negative breast cancers, especially the triple-negative breast cancers (TNBC) [26], which are the most aggressive, faster-growing, and highly metastatic [16, 27]. This report, however, does not include information on ER, PR, Ki67, and HER2 status of breast cancer patients since they were not readily available. This could be the subject of further study.

From the immunohistochemistry results (Table 3 and Figure 1), tissue sections from breast cancer patients tended to have elevated DAPK1 expression. Whereas 75% (24 out of 32) of the cancer tissues was positive for DAPK1, only 13% (4 out of 32) of the controls was positve. About 88% (28 out of 32) of the nonbreast cancer tissue samples stained negative for DAPK1 compared to the 25% (8 out of 32) of the cancer tissue samples. This suggests that, ordinarily, DAPK1 levels in cells and tissues may be quite low and thus only get elevated in the face of genomic insults or cellular perturbation. However, DAPK1 levels were relatively substantial in the sera of both breast cancer patients and the controls even though that of the controls was significantly lower than that of the breast cancer patients. The differences in DAPK1 levels in sera and tissue biopsies are worth noting since DAKP1 seems to be more abundant in circulation than in breast tissues. This improves its potential of being used as a noninvasive blood biomarker.

Of the 32 breast cancer cases, 1, 25, and 4 were of grade I, grade II, and grade III, respectively (Table 2) and 2 ungraded. All the 4 samples of grade III exhibited elevated DAPK1 expression. Two (2) of them showed moderate staining reaction (scored +2) with the other two (2) showing strong staining reaction (scored +3). The preponderant grade II tissue samples showed mild staining reaction (scored +1). In our previous report [23], the association between DAPK1 and tumour grades could not be established. With observations from this current study, there seems to be an association between the two where DAPK1 expression increases with increasing tumour grade. Concomitantly, 88% of the noncancer tissue samples showed no staining reaction (scored 0).

For the reliability of DAPK1 as a noninvasive biomarker, the effect of chemotherapy on serum DAPK1 expression was evaluated in breast cancer patients. A “*treatment*” group was established which had participants who were already receiving chemotherapy for breast cancer prior to their enrollment into the study while a “*pretreatment*” group had participants who were not on chemotherapy though diagnosed of breast cancer. Participants in the “*treatment*” group were already on medications such as Doxorubicin, Cyclophosphamide, Paclitaxel, Trastuzumab, and 5-Fluorouracil; drugs which aim at destroying tumour cells in tissues and metastasized cancerous cells in the blood through apoptosis [28, 29]. From Table 2, even though DAPK1 expression was slightly higher in the “*treatment*” group compared to the “*pretreatment*” group, the difference was not statistically significant. From the results, it appears that the chemotherapy drugs do not affect DAPK1 expression. This could be due to the quick degradation of the drugs by the body after their administration. Our observation corroborates the report of Lui and Chu [30] who compared promising noninvasive biomarkers in serum for the early detection of gastric cancer. In light of the above observation, DAPK1 holds potential as a cost-effective means of monitoring tumour burden in response to chemotherapy in breast cancer patients and further investigations in this direction will be beneficial.

In summary, augmented DAPK1 expression in cancers can be interpreted as a stress response survival mechanism to remediate ongoing deleterious events in the cells orchestrated by carcinogenesis. DAKP1 was highly expressed in samples of breast cancer patients than in controls. Comparing levels of expression, the protein was highly abundant in sera compared to breast tissue biopsies. Also, chemotherapy did not interfere with DAKP1 expression levels and therefore makes the protein quite stable and reliable. DAPK1 may have potential application as a blood biomarker for breast cancer as well as the prospect for developing new therapeutic strategies. If increased expression is associated with aggressive tumours, then, targeting DAPK1 could be a treatment method as well for curtailing tumour aggression and progression.

## 5. Conclusion

DAPK1 is highly expressed in sera and breast tissues of breast cancer patients than nonbreast cancer participants. The expression of the protein seems to have an association with tumour grades and thus increases with increasing tumour grade. Furthermore, DAPK1 levels are relatively substantial in sera than in breast tissue biopsies. The elevated expression of DAKP1 in circulation rather than in breast tissues makes it a candidate for use as a blood biomarker and holds prospect for developing new therapeutic strategies. With the observed increased expression of DAPK1 in association with breast cancer, the protein can well be a target for therapeutics and may potentially serve as a biomarker for tumour aggression and prognosis of the disease. Further studies in this direction will be insightful.

## Figures and Tables

**Table 1:** Demographics and clinical information of breast cancer patients and controls.

Parameter	Out of 32 breast cancer cases	Out of 32 controls
Occupation		
Government workers	7	11
Petty traders	10	5
Unemployed	15	4
Fashion industry/artisanship	-	10
Unskilled labour	-	2
Breast cancer grade		
Grade I	1	
Grade II	25	
Grade III	4	
Grade IV	0	
Grade X	2	
Diagnosis		
Invasive ductal carcinoma	30	
Metastatic carcinoma	2	

**Table 2:** Mean serum DAPK1 concentration among groups.

Group	Mean serum DAPK1 concentration (ng/ml)	*p* value
Breast cancer patients (cases)	4.95 (SD = 1.53)	
*Treatment* group	5.19 (SD = 1.95)	0.800^∗∗^
*Pretreatment* group	4.68 (SD = 1.90)	0.021^∗∗∗^
Healthy individuals (control)	3.49 (SD = 1.72)	0.039^∗^

Note: ∗ compares cases and controls; ∗∗ compares *treatment* and *pretreatment* groups; ∗∗∗ compared *treatment* and *control* groups.

**Table 3:** DAPK1 expression patterns in breast biopsies.

Group	DAPK1 expression intensity (no. of samples)				Total	*p* value
	0	+1	+2	+3		
Breast cancer	8	15	6	3	32	<0.001
Nonbreast cancer	28	4	0	0	32	

## Data Availability

All data generated and analyzed during this study are included in this published article.
